# Characterization of Conformational Instability of Monoclonal Antibodies During Chromatographic Purification

**DOI:** 10.3390/ijms27042064

**Published:** 2026-02-23

**Authors:** Krystian Baran, Rafał Podgórski

**Affiliations:** Department of Medicinal Chemistry and Metabolomics, Faculty of Medicine, University of Rzeszów, AI. Tadeusza Rejtana 16C, 35-959 Rzeszów, Poland; rpodgorski@ur.edu.pl

**Keywords:** monoclonal antibodies, biological drugs stability, aggregation, preparative chromatography

## Abstract

Monoclonal antibodies represent one of the fastest-growing sectors of the biopharmaceutical industry. Their high therapeutic efficacy and reduced incidence of adverse effects compared to conventional therapies have led to an increasing demand for these products. The costliest stages of monoclonal antibody production are the separation and purification processes, which underscores the need for continuous development and optimization of applied methodologies. Active pharmaceutical ingredients must exhibit high purity and preserved biological activity in order to meet stringent regulatory requirements. Macromolecules such as monoclonal antibodies possess complex conformational structures that significantly influence their stability. The application of multi-step chromatographic processes during purification from cell culture harvests may induce structural alterations, including protein unfolding and aggregation, ultimately resulting in decreased product quality and therapeutic effectiveness. Such structural changes may also increase immunogenicity risk and reduce product shelf life, posing additional challenges for downstream processing. In addition, chromatographic media create microenvironments that differ markedly from bulk solution (e.g., high local protein concentration, confined pore spaces and heterogeneous surface chemistry). These effects can promote either self-association driven by colloidal interactions or partial unfolding followed by irreversible aggregation, depending on the unit operation and operating window. Practical mitigation is therefore rarely achieved by a single lever; instead, it requires an integrated view of resin selection, buffer composition (pH, salt type and ionic strength, and stabilizing additives), residence time and temperature, as well as an analytics strategy that combines orthogonal aggregation assays with structural probes. This work discusses the phenomena of unfolding and aggregation of therapeutic proteins, with particular emphasis on monoclonal antibodies occurring during chromatographic purification. Furthermore, key analytical methods, characterization techniques, and mitigation strategies aimed at improving product quality and reducing manufacturing costs are reviewed.

## 1. Introduction

The biopharmaceutical sector is characterized by a high growth rate and strong demand for innovative therapeutics. Monoclonal antibodies (mAbs) production technology is a crucial and complex process in this sector, particularly for IgG antibodies, which account for the majority of marketed or late-stage mAbs-based drugs [1,2,3,4]. Their global market size is projected to reach USD 823.31 billion by 2034 [5].

The first step of therapeutic mAbs production—the Upstream Process (USP)—remains challenging due to issues with cell line stability, variable expression yields, and difficulties in controlling post-translational modifications, especially IgG glycosylation, which strongly affects antibody quality [6]. Even in well-established systems such as CHO cells, clone-to-clone variability and genetic or epigenetic drift may lead to inconsistent productivity over prolonged culture periods. Moreover, optimizing culture conditions (media composition, pH, nutrient feeding, oxygenation) is crucial, as slight variations can alter N-glycan profiles, impacting drug efficacy, clearance, and immunogenicity [7]. On the other hand, upstream processes of mammalian cell-culture therapeutic monoclonal antibody manufacture are now sufficiently understood to allow for predictive process modelling and reproducible scale-up. Recent reviews demonstrate that cell-line selection, media formulation, feeding strategies, and control of culture conditions have matured into a standardized toolbox that enables robust and reproducible upstream workflows [8].

The cell culture harvest obtained from the USP is subjected to protein isolation and purification in the subsequent downstream processing (DSP) stage. Impurities present in the post-fermentation mixtures can cause adverse effects in humans, making their removal a critical step in monoclonal antibody production. Due to high costs and stringent regulatory requirements for drug purity, the applied purification processes currently in use still require further optimization and improvement [9,10].

**Figure 1:**
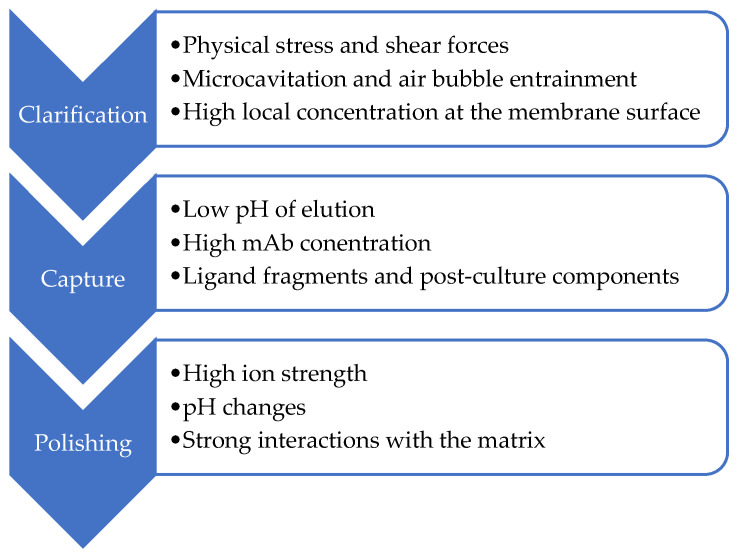
The main causes of monoclonal antibody instability during the purification process.

Typical DSP steps include clarification (centrifugation, filtration), capture (chromatography based on specific ligands, e.g., protein A or G), and a series of polishing steps, mainly ion exchange and hydrophobic or multimodal chromatography [9]. Across these operations, monoclonal antibodies are exposed to unfavorable conditions that promote conformational changes, leading to reduced efficiency and selectivity of separation in preparative chromatography (Figure 1).

Aggregation of monoclonal antibodies is a major quality concern because it affects both product stability and patient safety. Aggregates can reduce therapeutic efficacy by blocking antigen-binding sites or altering the antibody’s structure. More importantly, they can trigger immune responses, including anti-drug antibodies (ADAs), which may neutralize the treatment or cause adverse reactions [11]. Aggregation also compromises formulation stability and can shorten product shelf life. Therefore, strict control of aggregation is essential to ensure the safety and effectiveness of mAb-based therapies [12]. From a quality perspective, aggregates and particles are commonly treated as critical quality attributes (CQAs) that must be controlled through a combination of process design (preventing formation), process clearance (removing species that form), and robust analytical monitoring. Because chromatography is used repeatedly across the downstream process train, it can act both as a purification tool and as a stressor that shifts the aggregation landscape. A mechanistic understanding of when and why this happens is therefore essential for rational process development rather than trial-and-error optimization [13,14,15].

Pharmaceutical production requires exceptionally high product purity and reliable downstream operations, making chromatography a central operational unit in protein purification. Furthermore, chromatographic processes can impact protein structural stability. Adsorption-induced conformational changes often reduce separation selectivity and lead to yield losses in industrial applications. The stability of antibodies and other protein drugs is a widely studied process; nevertheless, the mechanisms underlying protein unfolding on chromatographic media remain only partially understood. This phenomenon results from complex kinetic and thermodynamic interactions dependent on numerous process parameters, making predictive modeling difficult. Furthermore, experimental challenges associated with real-time protein stability assessment limit the ability to monitor and control unfolding during chromatographic separation.

In this review, we summarize recent reports on conformational changes and aggregation of monoclonal antibodies during chromatographic purification. Mechanistic drivers of antibody destabilization during major chromatographic unit operations used in downstream processing—including affinity capture (Protein A), ion exchange chromatography, hydrophobic interaction chromatography, and multimodal chromatography—are discussed. The review emphasizes how the resin microenvironment, solution conditions, and process parameters shape conformational transitions and aggregation pathways, and practical analytical and mitigation strategies that can be implemented during process development and manufacturing are outlined. Particular attention is given to mechanisms driving structural destabilization and to analytical and process strategies for their detection and mitigation, supporting process optimization and cost reduction.

## 2. Review Methodology

This review was designed to capture evidence that is directly relevant to contemporary monoclonal antibody downstream processing and chromatographic purification. Accordingly, the search and screening strategy emphasized publications from 2000 to 2025, a timeframe that best reflects current platform processes, modern resin chemistries, and today’s analytical capabilities. To minimize the risk of overlooking influential work that may not be retrieved through keyword-based database queries alone, the reference lists of all eligible papers were also examined using backward citation tracking. Where needed, a small number of older, seminal publications were retained to provide essential mechanistic context, including foundational concepts in protein folding and aggregation and early descriptions of adsorption-driven conformational perturbations that still inform the interpretation of chromatographic behavior.

Studies were included only if they were published between 2000 and 2025, were available in English (when relevant, full texts could be accessed, Polish was also accepted), and could be retrieved as full-text articles via Google Scholar and/or PubMed or through publisher platforms and institutional access where necessary. To be eligible, a study had to address conformational instability, unfolding, self-association, and/or aggregation of monoclonal antibodies (IgG) or closely related therapeutic proteins specifically in the context of chromatographic purification. In addition, it needed to investigate at least one chromatographic unit operation commonly used in mAb downstream processing, such as affinity chromatography, ion exchange chromatography, hydrophobic interaction chromatography, or mixed-mode/multimodal chromatography, or focus on resin-related phenomena directly relevant to these operations. Eligible publications also had to describe or evaluate analytical tools capable of detecting structural changes or aggregation and/or propose or assess mitigation strategies aimed at reducing instability during chromatography. The evidence base was limited to peer-reviewed original research articles, systematic or narrative reviews, and method-focused papers; however, selected regulatory guidance documents and standards were also considered when they directly informed quality expectations or aggregation/immunogenicity risk assessment in biologics manufacturing.

Studies were excluded when their primary focus fell outside chromatography-driven structural effects, for example, when they addressed stability only in formulation contexts without a meaningful chromatographic linkage. Publications centered on small molecules or non-protein active pharmaceutical ingredients were also excluded unless they offered clear mechanistic insights transferable to mAbs or therapeutic proteins. Chromatography papers that reported separation performance alone, such as yield, purity, or resolution, without assessing or discussing conformational change, unfolding, self-association, or aggregation, were not considered relevant for this review. Non-peer-reviewed sources, including editorials, letters to the editor, opinion pieces, technical reports without peer review, and conference abstracts lacking full manuscripts, were excluded to maintain an appropriate evidence standard. Additional exclusions were applied when papers were not available in English or Polish, when full texts could not be accessed, or when methodological reporting was insufficient to interpret findings in the context of mAb conformational stability, for instance, if resin type or operating conditions were not specified or analytical readouts were unclear.

## 3. Unfolding and Aggregation of Monoclonal Antibodies

**Figure 2:**
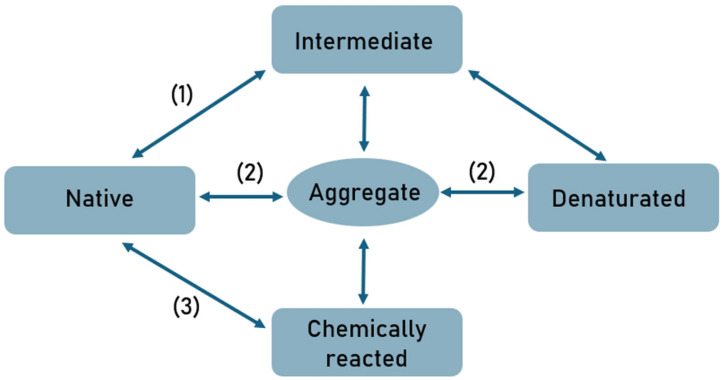
Classical protein instability pathways: (1) physical aggregation; (2) self-association or chemical cross-linking; (3) chemically induced aggregation (adapted from [16]).

Adsorption and desorption processes on chromatographic media often lead to changes in protein structure and, consequently, changes in their activity. Protein unfolding can be irreversible, and the aggregates formed are usually characterized by different biological activity compared to the molecules in their native form. Denaturation, as a reversible or irreversible change in protein structure, is undoubtedly a precursor to protein aggregation. Protein aggregation is a complex, multistep process driven by the dynamic equilibrium between native, partially unfolded, and fully unfolded conformations (Figure 2). Partially unfolded monomers are considered key precursors of aggregation, as they expose hydrophobic regions and flexible segments that mediate intermolecular contacts required for self-assembly [16].

Aggregation proceeds through multiple, often overlapping pathways. Native monomers may self-associate reversibly via complementary surface interactions to form small oligomers, which can evolve into irreversible aggregates through covalent modifications, such as disulfide bond formation. Additionally, nucleation-dependent and surface-induced mechanisms induce conformational changes in partially unfolded or destabilized monomers, increasing their propensity for aggregation. Across all mechanisms, hydrophobic interactions constitute the major driving force, with additional contributions from electrostatic and van der Waals forces. Initial soluble oligomers progressively exceed solubility limits and convert into insoluble aggregates, which may take the form of amorphous precipitates or highly ordered fibrillar structures. The competition among these pathways and their sensitivity to environmental factors highlights the multivariate nature of protein aggregation and underscores the importance of controlling solution conditions in both physiological and therapeutic contexts [16,17,18,19].

**Figure 3:**
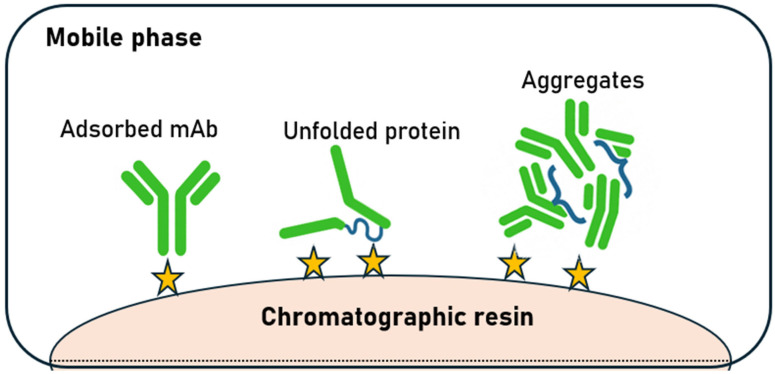
Hypothetical unfolding and aggregation pathway on chromatographic resins (yellow stars indicate ligands).

During the purification process, especially chromatography, hydrophobic domains are exposed, which are largely responsible for aggregation [20,21,22]. The aggregation process can be influenced by many factors, including temperature, protein concentration, pH, and solution ionicity. Furthermore, the presence of certain ligands, including specific ions, can stimulate changes in protein structure. Multidomain antibodies may also undergo domain swapping, leading to aggregates in which individual domains remain folded. In some cases, aggregation results from multiple overlapping physical mechanisms. During the production and purification of biopharmaceuticals, mechanical stresses also occur, which can affect product aggregation [17,23,24,25,26]. Depending on the process conditions, the protein binds in its native form or unfolds during adsorption onto the chromatographic medium, which can lead to the formation of second- or higher-order aggregates. The hypothetical unfolding and aggregation pathway on chromatographic resins is shown in Figure 3. It is hypothesized that the adsorbed protein, once anchored to the adsorbent surface, unfolds through interactions with several adjacent active sites. The unfolded protein is susceptible to aggregation [16].

Monoclonal antibodies are multi-domain proteins composed of Fab and Fc regions connected by a flexible hinge. Local stability is heterogeneous: certain domains may unfold earlier than others, and variable regions can contain exposed hydrophobic and charged patches that promote self-association or resin binding. Glycosylation of the Fc region, sequence-dependent surface charge distribution, and the presence of engineered mutations (e.g., for altered effector function or half-life extension) can further modulate both conformational and colloidal stability [11,27,28]. In chromatographic processes, these molecular characteristics govern the interaction strength between an antibody and a specific ligand or ion-exchange matrix, influence whether binding favors orientations that expose hydrophobic surfaces, and affect the ability of the molecule to regain its native conformation upon desorption. Consequently, generic “platform” purification conditions that perform well for most IgG1 antibodies may be inadequate for molecules with unusual variable regions or for structurally more complex formats, such as bispecific antibodies, Fc-fusion proteins, or antibody fragments.

mAb stability during downstream processing can be described along two axes. Conformational stability reflects the resistance of an antibody to structural disturbances (e.g., unfolding, domain swapping, or local rearrangements), whereas colloidal stability captures the propensity for protein-protein interactions that drive reversible self-association and, eventually, aggregation. Chromatographic steps can challenge both dimensions by combining high local concentrations with rapid changes in solution conditions and strong interactions with stationary phases [29,30].

Protein conformational changes are a limiting issue in the production of biopharmaceuticals, which is why many analytical methods exist to detect this phenomenon. There are several approaches to predicting the aggregation of antibodies and other therapeutic proteins, including both screening [31,32,33,34] and modeling-based approaches [35,36,37], but the greatest challenge is the assessment of conformational changes during adsorption in real time. Mechanistic diagnosis typically requires orthogonal analytics. Techniques sensitive to secondary/tertiary structure (e.g., CD, FTIR, DSC/DSF, HDX-MS) can indicate conformational change, while size and particle-based methods (e.g., SEC, light scattering, micro-flow imaging) quantify aggregation across size regimes. In-column and in-line spectroscopic approaches can further reveal transient events that may be missed by fraction-based analysis, enabling a closer link between process conditions and structural outcomes [38,39,40,41]. The methods used to analyze structural changes (Table 1) and aggregation (Table 2) during and after the adsorption/desorption process are summarized below.

Beyond experimental analytics, modeling approaches can assist in translating mechanistic understanding into actionable process design. Mechanistic models may couple adsorption equilibria, mass transfer and residence time with kinetic schemes for unfolding and aggregation. Data-driven models can complement these frameworks by learning relationships between sequence-derived features, formulation variables and process outcomes, provided that training data are representative and analytically consistent. In practice, hybrid strategies—mechanistic constraints combined with statistical or machine-learning components—are particularly attractive for rapidly screening operating windows and identifying high-risk regions for aggregation [35,36,61].

## 4. Chromatography in mAbs Purification Processes

**Figure 4:**
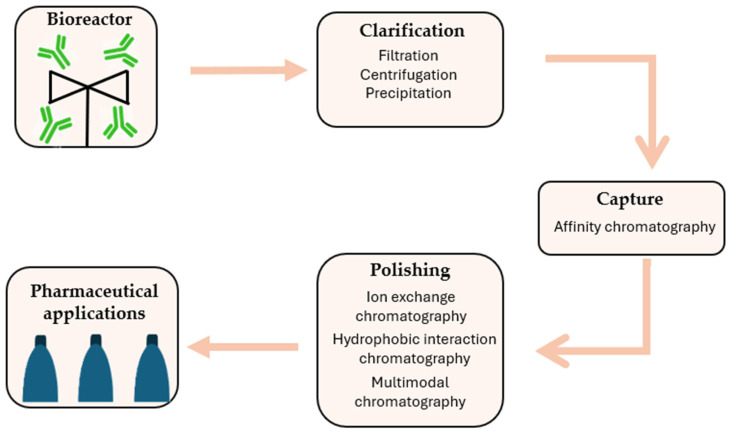
Typical mAb production process flow diagram.

Contemporary monoclonal antibody purification processes are increasingly designed around standardized platform approaches that rely on common unit operations. Nevertheless, variations in the physicochemical properties and behavior of individual antibodies make full standardization challenging. Consequently, depending on the specific characteristics of the antibody, the purification workflow typically incorporates multiple unit operations (Figure 4) [62].

The clarification stage, in particular, employs a combination of mechanical and chemical separation techniques, such as filtration, centrifugation, and precipitation, to effectively remove cells, contaminants, and process-related impurities [62,63]. Advances in monoclonal antibody development are moving toward higher cell culture densities, often accompanied by reduced cell viability, which impacts downstream purification processes, particularly operations involved in clarification and the removal of solid impurities [64].

Due to its high selectivity and efficiency, affinity chromatography has become an indispensable technique for the initial capture of target molecules. Bacterial-derived ligands, such as proteins A, G, and L, are most commonly employed due to their strong and specific binding to the Fc or Fab regions of antibodies. Nonetheless, their application is associated not only with significant costs but also with potential impacts on chemical stability and the risk of ligand leaching [65]. Novel peptide-based ligands, designed and optimized to target specific regions of antibodies, can maintain high performance while allowing mild elution conditions and exhibiting enhanced chemical stability [66,67]. An alternative may be offered by low-molecular-weight ligands, synthetic ligands, and advanced materials based on monoliths, chromatographic membranes, or nanomaterials. Nevertheless, despite the wide range of available ligands, protein A remains the most commonly used [68,69,70]. Desorption occurring during the affinity chromatography process, achieved under low pH conditions, contributes to the inactivation of potentially present viruses, including both RNA and DNA viruses, which is crucial for ensuring the biological safety of the final product [71].

Despite the high selectivity of product capture, it is necessary to employ methods that allow for the removal of trace impurities such as host cell proteins, DNA, endotoxins, residual ligands, and other process-related by-products that may remain after earlier purification steps. Chromatography, owing to its high selectivity and ability to precisely separate molecules with very similar physicochemical properties, constitutes a key tool for meeting the stringent quality standards required for biological products. Consequently, polishing steps enable the production of a highly pure final product, which is essential for pharmaceutical applications [9].

In the case of monoclonal antibodies and their increasingly diverse formats, the importance of chromatographic techniques in the polishing stage becomes even more pronounced. Differences in molecular architecture, surface charge distribution, hydrophobicity, and conformational stability influence antibody behavior under process conditions and determine separation efficiency. The application of appropriately selected chromatographic methods, such as ion-exchange, hydrophobic interaction, or mixed-mode chromatography, enables the selective removal of undesired product variants while preserving the structural integrity and biological activity of the antibody. In the context of the continuous development of therapeutic antibodies and the pursuit of more efficient and flexible manufacturing processes, chromatography remains an indispensable component of downstream processing, combining high separation performance with robust control of final product quality [9,10,62].

Chromatographic methods represent some of the costliest stages in the downstream processing of monoclonal antibody production, primarily due to the high price of chromatographic materials, limited resin lifetime, buffer consumption, and the complexity of equipment and process operations. A particularly significant cost driver is high-selectivity chromatography columns, which must meet stringent quality and regulatory requirements. Despite these economic constraints, chromatography often remains an indispensable component of mAb manufacturing, as it provides a unique capability to selectively remove impurities that are structurally and physicochemically similar to the target product, such as aggregates or charge variants. Consequently, even with extensive research into alternative purification strategies and process intensification, chromatographic techniques continue to form the cornerstone of ensuring high purity, safety, and regulatory compliance for therapeutic monoclonal antibodies [10,62,63].

### 4.1. Continuous Chromatographic Processes in mAbs Production

In recent years, the biotechnology sector has been rapidly advancing toward sophisticated chromatographic strategies that extend beyond traditional batch processes. A particular area of development involves semi-continuous and fully continuous processes, which integrate multiple chromatographic columns into logical operational sequences, enabling the elimination of downtime, more efficient utilization of chromatographic resins, and substantial enhancement of monoclonal antibody (mAb) purification performance [72]. Both semi-continuous and continuous chromatography approaches are employed in mAb production. Semi-continuous processes primarily include periodic counter-current chromatography (PCC), in which multiple columns operate cyclically, allowing an almost continuous feed of material and improved resin utilization. Continuous processes, on the other hand, include multicolumn countercurrent solvent gradient purification (MCSGP) and simulated moving bed (SMB) chromatography, which enable constant feed and product elution, thereby enhancing productivity, yield, and final mAb quality. This strategy aims not only to increase purification efficiency but also to improve the stability of chromatographed products and reduce operational costs, including buffer and resin consumption. These improvements are critical in the production of therapeutic monoclonal antibodies, where growing demands for scalability and product quality necessitate optimized downstream processes [72,73,74].

Moreover, continuous chromatography can influence the conformational changes of mAbs during the various purification steps. While gentler and more controlled loading and elution conditions can reduce structural stress on the protein, preserve its native conformation, and minimize aggregation, advanced continuous strategies also enable real-time monitoring of conformational variants. This allows for a better correlation between process conditions and the structural integrity of the product, which is essential for ensuring therapeutic efficacy and safety. Nevertheless, the design of semi-continuous and continuous processes requires a thorough understanding of the mechanisms of conformational changes and strategies to mitigate them at each stage.

### 4.2. Affinity Chromatography

Affinity chromatography is commonly used for the purification of monoclonal antibodies, but this process can induce both conformational changes and an increased tendency to aggregate. Studies indicate that direct contact of mAbs with affinity ligands can significantly accelerate aggregate formation, particularly during the subsequent desorption and low-pH viral inactivation step, suggesting a relationship between resin adsorption and antibody structural destabilization [75,76,77]. This effect arises because binding to the ligand can locally destabilize the mAb structure, transiently exposing hydrophobic regions that promote interactions with neighboring antibodies and aggregation. Transient conformational modifications of IgG during passage through the affinity column confirm that binding and elution alone can induce irreversible or partially reversible structural changes in the Fab and Fc regions [78,79]. Furthermore, after elution from the affinity column, the mAb-containing eluate is typically held at low pH (usually around 3.5–4.0) for a defined period as part of the virus inactivation step, which can further destabilize the protein’s structure [78,80].

**Figure 5:**
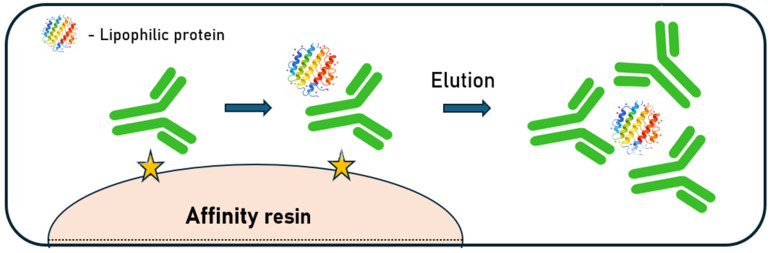
Proposed mechanism of mAbs destabilization by forming complexes with protein contaminants (based on [83]; yellow stars indicate ligands).

The mechanisms underlying aggregation are complex and can be attributed to a combination of specific and nonspecific interactions between the antibody surface and the immobilized Protein A ligand. Aggregated forms of monoclonal antibodies exhibit a higher affinity for the resin surface and tend to preferentially adsorb to the resin, which may further promote oligomerization of monomeric species in the eluate. Biophysical measurements show that low pH alone need not cause aggregation: a human IgG4 kept at pH 2.7–3.5 remained mainly monomeric by sedimentation velocity, retained near-UV CD structure, and displayed native-like DSC transitions. Aggregation emerged upon neutralization, yielding ~30% oligomers and incomplete spectral recovery, consistent with kinetic trapping during the pH shift [81]. In addition to antibody–ligand interactions, nonspecific impurities present in the harvested material, such as chromatin fragments and host-cell proteins (HCPs), may further influence aggregation behavior. These contaminants can stabilize pre-existing aggregates or promote de novo aggregate formation through electrostatic and hydrophobic interactions with both IgG molecules and the Protein A ligand [82]. Furthermore, binding to Protein A may expose hydrophobic regions that drive association with lipophilic host-cell proteins. Confocal and electron microscopy revealed intraparticle accumulation only in the presence of antibody, with co-localization of the mAb, bovine serum albumin, and α-lactalbumin. These mixed complexes can partially refold after low-pH elution but progressively deposit as aggregates over repeated cycles without stringent cleaning (Figure 5) [83].

Protein A-derived or co-eluting species can promote antibody aggregation, but upstream chromatin-directed clarification reduces histones, DNA, host-cell proteins, and aggregates while minimizing column fouling [84]. Studies on protein stabilization have shown that the addition of certain amino acids, particularly arginine, can effectively limit aggregate formation during protein refolding, suggesting a potential strategy for modifying elution buffers in affinity chromatography [85]. Improving adsorption and stability on affinity resins reduces structural stress and aggregation of IgG1 antibodies, which transiently adopt a reduced-size conformation (~5.5 nm) during Protein A elution that is reversible under physiological conditions but more susceptible to secondary stress [86].

Differences in antibody variable regions can determine their interactions with affinity ligands, which affects both purification efficiency and the risk of aggregate formation. Structural analysis revealed that H1 and H2 antibody dimers differ in Fab–Fab and Fc–Fc interactions, explaining their distinct Protein A binding and providing mechanistic insight into HMW removal during purification [87]. Optimizing operational parameters, including elution pH, resin contact time, and the use of stabilizing buffers, can significantly reduce the risk of structural degradation and aggregation during affinity chromatography [88,89].

Thus, although affinity chromatography offers high selectivity, it can induce transient or permanent conformational changes in mAbs and promote aggregate formation, requiring careful process optimization and consideration of stabilizers or additional polishing steps. Practical mitigation options include minimizing time at low pH, employing gentler elution profiles (e.g., controlled gradients rather than sharp steps when feasible), optimizing temperature, and using buffer systems or additives that stabilize the antibody during the pH transition. Importantly, neutralization itself is a mixing event that can create local pH gradients; controlled addition and adequate mixing can therefore reduce transient exposure to destabilizing microenvironments. Furthermore, intensive cleaning procedures may reduce bioburden risk but can also influence ligand stability and leachables, whereas insufficient cleaning can increase fouling-driven heterogeneity.

### 4.3. Hydrophobic Interaction Chromatography

**Figure 6:**
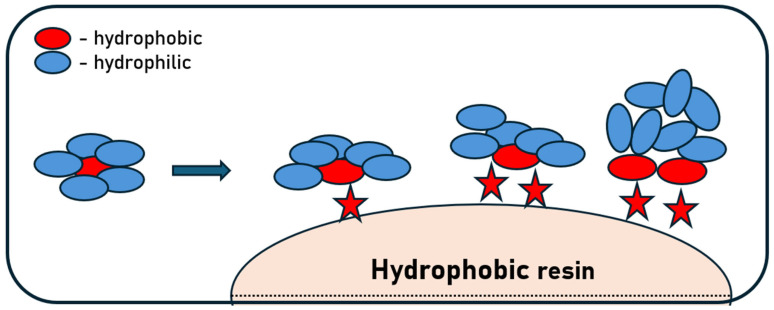
Mechanism of exposing hydrophobic fragments and unfolding proteins during adsorption (stars indicate hydrophobic ligands).

In the processes of separation and purification of monoclonal antibodies, hydrophobic interaction chromatography (HIC) is a widely used and significant technique. It relies on the hydrophobic properties of recombinant antibodies through interactions between non-polar regions on their surface and hydrophobic ligands immobilized on the chromatography matrix. mAbs are characterized by a well-defined number and a variable degree of exposure of hydrophobic groups on their surface. These subtle structural differences result in distinct interactions of individual mAb molecules with their surrounding environment. In the presence of kosmotropic salts, which influence the ordering of water molecules and enhance hydrophobic interactions, these differences become particularly pronounced. By applying an appropriately selected range of kosmotropic salt concentrations, it is possible to selectively modulate the behavior of individual antibodies, thereby enabling their effective separation. However, hydrophobic segments of monoclonal antibodies are most often located in the internal part of the protein structure, where they contribute to maintaining proper folding and structural stability. Consequently, during purification by HIC, exposure of these hydrophobic regions to the stationary phase may induce partial unfolding or structural rearrangements of the antibody molecules, leading to conformational changes that can affect their stability and functional properties (Figure 6) [90,91,92,93]. Protein unfolding on the matrix is largely a reversible phenomenon; nevertheless, aggregate formation remains one of the major limitations of HIC. Hydrophobic interactions in HIC are not solely passive—partial unfolding of proteins, increased mobility of molecular segments, and transient exposure of hydrophobic domains occur, which enhances the affinity both to the stationary phase and to other protein molecules [94,95,96].

Studies on protein interactions with HIC matrices clearly demonstrate that both the extent of retention and the efficiency of protein recovery are governed by a complex interplay between the intrinsic properties of the protein and the characteristics of the applied sorbent. Key protein-related factors include molecular weight, structural flexibility, and tertiary and quaternary conformations, which collectively influence the accessibility and dynamic reorganization of hydrophobic regions during the adsorption process. At the same time, sorbent properties such as porosity, ligand density, and the chemical nature of the hydrophobic ligand, as well as the type and rigidity of the support matrix, determine the strength and mechanism of protein–resin interactions. The characteristics of the matrix significantly influence mAb stability—highly hydrophobic ligands improve selectivity but also elevate the risk of denaturation, whereas adsorbents with moderate interaction strength reduce structural changes at the expense of separation efficiency [97,98]. Dynamic binding capacity and mass transfer kinetics determine the efficiency of adsorption. Diffusional limitations within the sorbent pores may promote prolonged contact between the protein and the surface, increasing the probability of unfolding and aggregation [99]. Such extended surface interactions may promote conformational rearrangements that expose additional hydrophobic regions, further strengthening protein–sorbent interactions. HX-MS studies of α-lactalbumin have shown that adsorption to HIC media can selectively affect native and unfolded species, with increased labeling indicating surface-induced unfolding. Salt concentration modulates this effect: at intermediate ammonium sulfate levels, the surface-induced destabilization is partially mitigated, while, in the absence of salt, unfolded species adsorb more strongly and are further destabilized [100]. The elution stage also carries a risk of destabilization—abrupt changes in pH or ionic strength may prevent complete refolding to the native state, stabilizing partially unfolded structures and highlighting the need to consider both ligand type and mobile-phase conditions when developing HIC processes [95]. Moreover, protein spreading on the stationary phase enhances hydrophobic contacts with the chromatographic resin, which can facilitate aggregation or irreversible binding of proteins. HIC adsorption should be considered a multi-stage process, and performing it at high salt concentrations can decouple primary adsorption from spreading, preserving protein stability and reducing undesired structural changes [101].

The selection of appropriate process conditions often allows control over unfavorable conformational effects that may impact final product quality and increase overall process costs. Mitigation strategies in HIC often focus on reducing the combined stresses of high salt and strong hydrophobic adsorption. Options include using fewer hydrophobic ligands, operating at the lowest effective salt concentration, selecting salt types that balance selectivity and solubility, applying gradients rather than step changes, and controlling temperature. Additives that reduce protein–protein interactions or stabilize native structure can also be evaluated, but their impact on chromatographic selectivity must be considered [92,94,98].

### 4.4. Ion Exchange Chromatography

Ion exchange chromatography (IEX) is based on electrostatic interactions and is one of the key techniques for protein separation in bioprocesses. Anion exchange chromatography is primarily used to remove anion-based contaminants (host-cell proteins, DNA, endotoxins, protein A residues), while positively charged antibodies flow through the chromatographic column [102,103,104]. Cation exchange chromatography (CEX) exploits the positive charge of mAbs at a specific pH, allowing them to bind to a cation exchanger, and elution allows for the separation of often slightly different contaminants (charge variants, aggregates, proteins with similar structures) [102,105,106].

The process of adsorption and desorption on the substrate surface affects the structure and stability of mAbs. Electrostatic interactions, characteristic of IEX, can lead to subtle or profound conformational changes and, consequently, aggregate formation. Protein binding to ionic ligands occurs under conditions of forced contact with the surface and local charge gradients, which can modulate the equilibrium between the native structure and partially unfolded states. Such surface-induced perturbations may alter intramolecular electrostatic networks and expose aggregation-prone regions [107,108]. Based on a comparative analysis of different ligands acting on adsorbed Bovine serum albumin, it was demonstrated that the denaturation pathway is not governed solely by electrostatic interactions but is also strongly influenced by the chemical nature of the ligand and the additional interaction mechanisms arising from it [109].

Conformational change and aggregation phenomena are strongly dependent on pH, buffer concentration, protein mass, and flow rate. Enhanced protein unfolding was observed on polymer-grafted resins or resins with small pore sizes, whereas on conventional macroporous resins lacking polymeric extenders, this phenomenon was substantially attenuated or entirely absent [110]. Conditions favoring strong binding (e.g., low pH, low buffer concentration, and lower protein mass load) increase the likelihood of protein unfolding and formation of aggregates, as observed for glycosylated IgG_2_ on Fractogel CEX columns. Higher salt and buffer concentrations, on the other hand, reduce the strength of the interaction, limiting undesirable structural changes and complex elution profiles [111]. Protein-resin interactions during adsorption on IEX resins often result in additional chromatographic peaks resulting from the formation of a strongly bound, partially aggregated form of the mAb [51,111].

Multi-peak elution on cation exchange resins arises from binding sites with different strengths and kinetics. For IgG_2_ on POROS XS, the first two peaks contain monomers, while a third peak with aggregates grows with hold time, reflecting unfolding at strong, slow-binding sites [112]. For human serum albumin, two peaks contain monomeric and dimeric species, with the second peak affected by flow rate and temperature, consistent with a kinetic limitation or distinct adsorption conformations [113]. Overall, prolonged binding to high-affinity sites promotes unfolding and aggregation, whereas weaker sites retain native monomers, and adjusting load buffer conditions, such as adding arginine, can mitigate aggregation [112,113]. The observed effects in CEX chromatography strongly depend on pH, temperature, residence time, salt concentration, and modifiers. Histidine residues in CDRs can induce double-peak elution [114], low salt slows partially unfolded IgG and increases retention [115], and a glycosylated antibodies show increased HMW formation and atypical peaks that can be mitigated by arginine, glycine, or citrate [116]. High NaCl concentrations promote reversible self-association of mAb, increasing the size of later elution peaks, while chaotropes and positively charged amino acids, especially histidine, reduce this effect, likely by modulating protein–resin interactions, shifting elution to lower NaCl concentrations [117].

**Figure 7:**
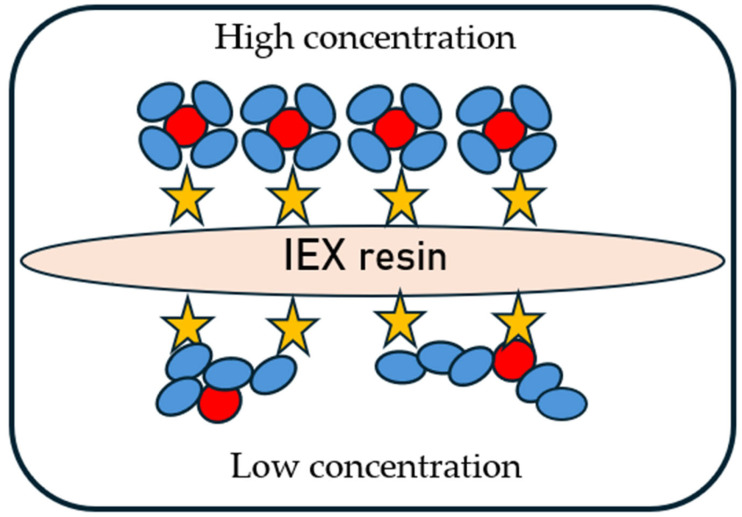
Hypothetical crowding effect on the surface of IEX resin (stars indicate ligands).

The phenomenon of antibody unfolding and aggregation may depend on the parameters of the chromatographic resin used, e.g., matrix, functional group, particle size, and ligand density. Furthermore, the use of higher protein concentrations and column overloading can improve the stability of the adsorbed molecules. This is explained by the so-called crowding effect at high surface coverage, where a compact layer of adsorbed proteins restricts unfolding, thereby reducing aggregation and enhancing protein recovery (Figure 7). This effect has also been observed previously on HIC resins [118] and is relevant for mAb2 and mAb3 in CEX chromatography [34].

Protein adsorption on IEX resins is a complex process involving not only electrostatic interactions but also conformational reorganization, destabilization of the native structure, and the formation of intermediate states predisposed to aggregation. Common mitigation levers include decreasing load density, shortening residence time, optimizing pH to balance binding strength and conformational stability, and selecting resins with appropriate pore structure and ligand chemistry. Buffer species and additives can modulate both protein–resin and protein–protein interactions; however, they may also change selectivity and must be screened with chromatographic performance. Because IEX is frequently used late in the purification train, its stability impact can be amplified: aggregates formed at this stage may be difficult to remove downstream, underscoring the value of early detection and conservative operating windows [111,116].

### 4.5. Multimodal Chromatography

Multimodal chromatography (MMC) in antibody purification utilizes ligands that interact with the molecule being separated through two or more mechanisms, based on simultaneous or independent electrostatic, hydrophobic, and hydrogen interactions, resulting in high separation selectivity [119,120,121]. In the case of biological drugs, the ability to control multiple variables on the one hand provides broader application and flexibility in method selection, but, on the other hand, it also generates an increase in potentially undesirable phenomena affecting the quality of the separated components [122,123]. During purification using MMC, similarly to IEX and HIC, conformational changes, protein unfolding, and aggregation can occur [109].

Destabilization of protein structure after binding to the resin surface leads to partial unfolding of the molecules and initiates aggregation, a phenomenon particularly pronounced on more hydrophobic multimodal resins. Experimental observations showed that BSA monomer formed dimers and higher-order oligomers on multimodal anion exchangers, whereas binding to a more hydrophilic monomodal resin resulted in minimal aggregation even after prolonged incubation [124]. On overloaded multimodal cation exchangers, broad, highly tailing elution peaks and on-column formation of aggregated species were observed, especially under conditions of strong binding (low ionic strength), while monomodal resins yielded sharp peaks and high recoveries [125]. Conditions promoting stronger binding—low ionic strength, high pH, and elevated temperature—further increased aggregate formation, reducing yield and recovery of separated substances. Conversely, higher salt concentrations and lower surface loading mitigated on-column aggregation and improved protein recovery [124,125,126]. In addition to standard process parameters, structural changes can be induced by specific protein–protein interactions, which are enhanced at high adsorbent surface coverage. Studying the adsorption of model proteins and mAb showed that high surface coverage enhanced cooperative interactions, affecting adsorption equilibria and isotherm shapes, although under mild adsorption conditions, these effects remained reversible and did not induce aggregation or structural destabilization [127]. One way to reduce the undesirable phenomenon of unfolding and aggregation is to utilize arginine in the separation process. Guanidine groups enable interactions with both aromatic residues of proteins and ligands immobilized on the bed, which weakens the undesirable interactions responsible for structural changes [128].

Mixed-mode functionalities are increasingly implemented not only on traditional bead resins but also on membranes and monoliths. These formats can reduce diffusion limitations and shorten residence times, potentially decreasing time-dependent unfolding. They also enable high-throughput polishing in single-use setups, which may reduce cross-contamination risk and simplify changeover. However, the altered hydrodynamics and surface-to-volume ratio can change the extent of surface exposure, again motivating molecule-specific evaluation of stability during adsorption and elution [129,130].

## 5. Manufacturing Quality Control and Mitigation Strategies

While each chromatographic mode relies on its own dominant interaction mechanisms, many of the practical ways to protect antibodies from damage are shared across platforms. In most development programs, building a robust control strategy means doing more than choosing the “right” resin or buffer. It requires coordinating material selection with tight control of operating conditions, such as load, residence time, gradient shape, and temperature, and pairing these choices with at-line or in-line analytics that can detect early signs of destabilization.

During process development, teams typically focus on a handful of recurring levers. First, they try to limit exposure to extreme pH conditions and, just as importantly, to avoid abrupt pH shifts, particularly during low-pH elution and neutralization, where pH shocks can be most pronounced [131,132]. In parallel, protein concentration and resin contact time are managed to reduce crowding effects and minimize surface-induced unfolding events [112,116,133]. Ionic strength is then tuned, often together with salt identity and buffer composition, to weaken unwanted electrostatic or hydrophobic interactions that can promote retention anomalies or structural perturbations [117,134,135].

Because interfaces and flow-induced forces can also contribute to instability, developers frequently aim to reduce interfacial stress (for example, by preventing air entrainment and foaming) and moderate mechanical stress during pumping and mixing [136,137,138]. On the materials side, resin chemistry, ligand density, and pore architecture are selected to reduce secondary interactions and lower the risk of irreversible adsorption [139,140,141]. Finally, when compatible with product requirements and downstream clearance, stabilizing additives or excipients (e.g., arginine or histidine) may be introduced to improve conformational stability and suppress aggregation tendencies [142].

Understanding the mechanisms of therapeutic protein destabilization is essential for process optimization and ensuring drug safety. Monitoring and assessing structural changes and aggregation are covered by the FDA regulatory guidelines [143]. Altered protein structural stability, leading to unfolding, denaturation, or aggregation, can induce an immune response to drugs. Soluble and insoluble protein aggregates are considered potentially potent immunogenic factors, potentially facilitating antigen uptake by cells and influencing immune activation. FDA guidelines describe the need for monitoring at various stages of the product life cycle, including production, storage, and transportation. A range of experimental methods are necessary to determine the contribution and impact of aggregates on the safety and efficacy of therapy. This approach should be based on optimizing process conditions through empirical and predictive approaches.

The complex structure of therapeutic proteins, the multitude of process parameters, and the dynamics of the chromatographic processes make predicting structural changes and their impact on the final product stability very difficult. One approach is the use of high-throughput screening, which allows for the screening of a large number of candidate protein drugs or the selection of chromatographic media that do not negatively impact protein structure [34]. Models based on molecular dynamics, machine learning, and artificial intelligence are increasingly used [37,61,144], but predicting structural changes during the adsorption/desorption process is difficult to design. Simplified models of adsorption dynamics during chromatography have been implemented in modeling separation using typical chromatographic techniques, but they do not account for the competing interactions of transient species and aggregates formed during protein unfolding on the adsorbent surface. The development of reliable models requires a large amount of experimental data and their correlation with process parameters and variables [101,145,146,147].

## 6. Conclusions

Biopharmaceutical products based on monoclonal antibodies must be characterized by high quality and purity to ensure safety and eliminate adverse effects. The purification process for monoclonal antibodies relies primarily on chromatographic methods, which, although among the most expensive steps in the process, are highly effective and efficient, and usually cannot be omitted or replaced by other methods. Separation and purification based on adsorption on chromatographic resins can result in destabilization of antibodies and therapeutic proteins. The unfolding and aggregation of individual molecules into dimers and oligomers, which are higher-order aggregates, result in the formation of biologically inactive structures, the presence of which in the drug can cause undesirable side effects.

Separation methods, process conditions, and the materials used, such as chromatographic media, are adapted and optimized depending on the individual properties of the protein product. In addition to high separation selectivity, a key criterion should be the limitation of unfavorable structural changes in the protein. The presence of numerous modifications to chromatographic column packings on the market necessitates the development of efficient screening to quickly select the appropriate media. Another approach could be to use adsorption process modeling, which, however, should be correlated with empirical data.

Future downstream processes are likely to increase the use of continuous operations, membrane/monolith formats, and advanced analytics that move structural information closer to real-time. At the same time, the pipeline of antibody formats is diversifying, increasing the need for molecule-specific stability assessment rather than one-size-fits-all platform conditions. Open challenges include translating in-column structural observations into predictive process models, establishing broadly applicable stability metrics that correlate with clinically relevant aggregate classes, and developing robust, scalable mitigation strategies that preserve both product quality and manufacturing efficiency.

To date, research potential regarding the phenomenon of monoclonal antibody unfolding and aggregation during purification does not clearly explain this mechanism. The phenomenon is based on complex kinetic and thermodynamic effects, and the adsorption and chromatographic processes depend on numerous operational parameters, making it difficult to predict and describe.

## Figures and Tables

**Table 1:** Typical methods for studying conformational changes resulting from adsorption.

Method	Description	References
Circular dichroism	Measurement of the difference in circularly polarized light absorption (L/R) allows the assessment of secondary and tertiary structure.	[38,42,43]
Infrared spectroscopy	Analysis of amide bands allows the determination of the secondary structure of proteins.	[39,40,44,45]
Differential Scanning Calorimetry	Changes in protein melting temperature (Tm), indicating conformational destabilization.	[46,47,48]
Differential Scanning Fluorimetry	Use of hydrophobic dyes and measurement of melting temperature (Tm).	[34,41]
Deuterium-Hydrogen exchange combined with mass spectrometry	Exchange of hydrogen atoms for deuterium and analysis using mass spectrometry; the number and rate of exchanged atoms depend on the protein’s degree of folding.	[49,50,51]
Confocal microscopy	Visualization of protein structural changes.	[52]

**Table 2:** Typical methods for studying aggregation resulting from adsorption.

Method	Description	References
Size Exclusion Chromatography	Separation based on particle size on a column packed with a porous stationary phase; larger molecules migrate faster. UV detection enables determination of the percentage contribution of aggregates, while Multi-Angle Light Scattering allows the determination of particle size and molar mass.	[53,54]
Dynamic Light Scattering	Analysis of light-intensity fluctuations arising from Brownian motion enables calculation of the diffusion coefficient and the hydrodynamic diameter of the particles.	[55,56]
Analytical Ultracentrifugation	Centrifugal force with real-time monitoring of sedimentation enables precise determination of molar mass and molecular shape without labels or matrices.	[57,58]
Electrophoresis	Migration of charged molecules in an electric field; mobility depends on size, shape, and charge. Enables separation and quantification of aggregates, fragments, and monomers.	[58,59]
Micro-Flow Imaging	Protein sample flows through a measurement cell where particles are individually imaged; particle number, size, and morphology are determined from the images.	[60]

## Data Availability

No new data were created or analyzed in this study.

## Abbreviations

The following abbreviations are used in this manuscript:

mAbs	Monoclonal antibodies
IgG	Immunoglobulin G
CHO	Chinese hamster ovary
USP	Upstream process
DSP	Downstream process
ADAs	Anti-drug antibodies
AC	Affinity chromatography
HIC	Hydrophobic interaction chromatography
IEX	Ion exchange chromatography
CEX	Cation exchange chromatography
AEX	Anion exchange chromatography
MMC	Multimodal chromatography
SEC	Size exclusion chromatography
PCC	Periodic countercurrent chromatography
MCSGP	Multicolumn countercurrent solvent gradient purification
SMB	Simulated moving bed
CD	Circular dichroism
FTIR	Fourier transform infrared spectroscopy
DSC	Differential scanning calorimetry
DSF	Differential scanning fluorimetry
HDX-MS	Deuterium-Hydrogen exchange combined with mass spectrometry
DLS	Dynamic Light Scattering
AUC	Analytical Ultracentrifugation
MFI	Micro-Flow Imaging
HMW	High Molecular Weight
CDRs	Complementarity-Determining Regions
FDA	Food and Drug Administration
